# Cytomegalovirus in Bronchoalveolar Lavage Fluid: A Predictor of Outcome in Immunocompromised Patients

**DOI:** 10.1002/jmv.71163

**Published:** 2026-09-28

**Authors:** Theodora Zouvani, Meropi Karakioulaki, Kathleen Jahn, Sebastian Fähndrich, Björn C. Frye, Leticia Grize, Eleni Papakonstantinou, Hartmut Hengel, Karoline Leuzinger, Hans H. Hirsch, Daiana Stolz

**Affiliations:** ^1^ Clinic of Pneumology Medical Center – University of Freiburg Freiburg Germany; ^2^ Faculty of Medicine University of Freiburg Freiburg Germany; ^3^ Clinic of Dermatology Medical Center – University of Freiburg Freiburg Germany; ^4^ Clinic of Respiratory Medicine and Pulmonary Cell Research, University Hospital Basel Switzerland; ^5^ Freiburg University Medical Center, Faculty of Medicine, Institute of Virology University of Freiburg Freiburg Germany; ^6^ Clinical Virology, Laboratory Medicine University Hospital Basel Basel Switzerland; ^7^ Transplantation & Clinical Virology, Department Biomedicine University of Basel Basel Switzerland; ^8^ Division of Infectious Diseases & Hospital Epidemiology University Hospital Basel Basel Switzerland

## Abstract

Cytomegalovirus (CMV) is a prevalent herpesvirus that remains typically asymptomatic in immunocompetent individuals but can cause severe, potentially life‐threatening disease in immunocompromised patients. This study assessed the impact of CMV‐associated lower respiratory tract infections (LRTIs), diagnosed by polymerase chain reaction (PCR) testing of bronchoalveolar lavage fluid (BALF), focusing on coinfections and clinical outcomes. Between 2009 and 2017, a total of 2666 visits involving 1301 immunocompromised patients with suspected LRTI who underwent diagnostic bronchoalveolar lavage (BAL) were analyzed. The primary outcomes included CMV detection in BAL and resulting treatment modifications. Among these, 235 BALFs from 157 patients tested positive for CMV. Among these CMV‐positive cases, immunosuppression was classified as hematological conditions (*n* = 88), solid organ transplantation (*n* = 70), or other causes of immunosuppression (*n* = 77). Overall, 59 cases exhibited isolated CMV infection, which was associated with a higher percentage of macrophages in BALF, compared to cases with CMV and coinfection [75.5%, 95% confidence interval (CI): 44.0–85.0 vs. 37.5%, 95% CI: 11.5%–67.0%, *p* = 0.008]. A total of 176 cases demonstrated CMV and coinfection, which were associated with neutrophilia in BALF (49.5%, 95% CI: 15.5–82.5; vs. CMV‐monoinfection: 7.5%, 95% CI:2–36, *p* = 0.002). BAL findings prompted treatment modifications in 61.7% (*n* = 145) of all CMV‐positive cases. The 30‐day mortality rate was 13.6%, with a significantly elevated hazard ratio for those with CMV‐positive BAL diagnosis (unadjusted HR 2.85, 95% CI: 1.86–4.36). Cases with a CMV infection exhibited a significantly higher probability of requiring hospitalization compared to CMV‐negative cases, (OR 1.39, 95% CI: 1.03–1.87, *p* = 0.030). Immunocompromised patients have an increased risk of CMV infection, and the presence of CMV does not exclude a coinfection with other respiratory viruses, bacteria, or fungi. CMV detection in BALF was associated with higher hospitalization rates and mortality. Preventive and preemptive antiviral strategies are crucial, especially given the adverse impact of coinfections.

## 1 Introduction

In recent years, there has been a significant increase in the number of immunocompromised patients. Advances in medical treatments and technologies, such as organ transplantation, hematopoietic cell transplantation (HCT), and the management of autoimmune diseases, have improved patient survival rates but have also led to more patients with compromised immune systems [1, 2, 3]. This increase is also attributed to the expanded use of immunosuppressive therapies, such as corticosteroids and other immunomodulatory therapies, which are essential for managing chronic conditions but also reduce the body's ability to control infections.

The majority of lower respiratory tract infections (LRTIs) are caused by community‐acquired respiratory viruses, such as influenza and respiratory syncytial virus (RSV) [4]. However, nonrespiratory opportunistic viruses, such as cytomegalovirus (CMV) and herpes simplex virus (HSV), as well as other pathogens, may also play significant roles. LRTIs are a major health concern for immunocompromised individuals, who are more susceptible to these infections due to their weakened immune systems. Moreover, immunocompromised patients are at an increased risk for severe LRTIs caused by bacterial, fungal, and parasitic pathogens, which can lead to serious complications and increased mortality [3].

CMV, a ubiquitous herpesvirus, poses a significant threat to immunocompromised individuals. While CMV latency is tightly controlled in the immunocompetent hosts, CMV reactivation in those with weakened immune systems can easily progress to severe and potentially life‐threatening disease [5]. CMV serostatus indicates prior exposure to the virus, and seropositive individuals remain at risk for viral reactivation. Among the various manifestations of CMV in these patients, LRTIs are particularly concerning due to their high morbidity and mortality rates [6, 7].

CMV infection and CMV disease represent two distinct clinical entities, though they often present in overlapping ways, particularly in immunocompromised individuals. CMV infection refers to the presence of low‐level virus replication in the body, typically detected through PCR testing or viral cultures, and may be asymptomatic or cause mild, nonspecific symptoms [8]. In contrast, CMV disease refers to clinically significant manifestations of end‐organ disease, such as gastrointestinal disease, hepatitis, retinitis, or pneumonitis, often in the setting of immunosuppression [9, 10]. Distinguishing between CMV infection and CMV disease can be challenging in clinical practice due to the nonspecific nature of symptoms and the lack of clear‐cut diagnostic criteria. Asymptomatic CMV infection is common in immunocompromised patients, and the mere detection of CMV DNA does not necessarily imply infectious virus or active disease. Moreover, the presence of CMV can be confounded by other infections or coexisting conditions, complicating decision‐making regarding treatment [10]. Accurate differentiation typically requires a combination of clinical judgment, symptom assessment, imaging studies, and monitoring of CMV DNA loads, but even then, the diagnosis remains difficult and often requires careful consideration of the patient's overall immune status and risk factors.

Accurate and timely diagnosis of CMV‐related respiratory infections in immunocompromised patients is crucial for effective management. Fiberoptic bronchoscopy and the collection of bronchoalveolar lavage (BAL) samples is a key diagnostic tool in this context [11, 12]. Through techniques such as polymerase chain reaction (PCR), viral culture, and immunohistochemistry, CMV can be detected in BAL fluid (BALF), aiding in the timely initiation of antiviral therapy [4]. Given the complex clinical presentations and the overlap with other opportunistic infections, bronchoscopy plays a vital role in differentiating CMV from other pathogens, thereby guiding targeted treatment strategies.

This study represents one of the largest analysis conducted in immunocompromised patients, incorporating clinical, laboratory, and imaging data. Here, we aimed to evaluate the impact of CMV, diagnosed via PCR in BAL samples, on clinical outcomes in immunocompromised patients, while also considering coinfections. Specifically, the objectives are to determine the incidence of CMV detection in BAL samples from immunocompromised patients and to assess the clinical outcomes resulting from changes in patient management associated with this detection. By addressing these objectives, the study aims to evaluate the role of BAL in improving CMV diagnostic and therapeutic strategies.

## 2 Materials and Methods

### 2.1 Study Subjects

This is a prospective, single‐center study performed at the University Hospital Basel in Switzerland, with approval from the Institutional Review Board (EKBB05/06). Eligible participants were immunocompromised patients who underwent diagnostic flexible bronchoscopy with BAL between October 2009 and September 2017. Results reporting respiratory viruses by immunocompromised patients have been previously published [4]. Inclusion criteria comprised individuals aged 18 years or older presenting with suspected LRTIs, characterized by new or worsening respiratory and/or systemic symptoms (such as cough, sputum production, breathlessness), clinical signs (such as fever, tachypnea, abnormal pulmonary auscultation), and/or new or progressive radiological abnormalities. Patients were considered immunocompromised if they met any of the following criteria: history of solid organ transplantation or HCT, undergoing chemotherapy, progressive uncontrolled malignancy, untreated human immunodeficiency virus (HIV) infection, prednisone dosage exceeding 10 mg/day for over 4 weeks or receiving immunosuppressive treatment for interstitial lung or rheumatological diseases. Informed consent was obtained from all participants. Exclusion criteria included emergency cases where informed consent could not be obtained. Patients underwent routine clinical and laboratory examinations, with chest X‐ray and/or chest computed tomography (CT) scans if indicated by the attending physician.

### 2.2 Bronchoscopy With Bronchoalveolar Lavage

For all the bronchoscopies analgosedation with propofol and hydrocodone was used [13, 14, 15]. Lidocaine 1% was administered in 5 mL increments over the vocal cords, in the trachea, and main bronchi. Fiberoptic bronchoscopy was carried out via nose or mouth with patients positioned in a semi‐upright posture [16]. BAL was standardized by instilling 50 mL of sterile NaCl 0.9% up to three times through the working channel of the bronchoscope, targeting the affected lobe or segment as indicated by radiological findings. In cases of diffuse processes, the middle lobe was selected for lavage. The collected fluid was aspirated using suction.

### 2.3 Diagnostic Definitions

Patients were categorized into three groups: those with (a) hematological conditions, including a history of HCT or hematological malignancies, those with (b) a history of solid organ transplantation, and (c) others, including, but not limited, to patients with rheumatoid arthritis, interstitial lung disease, and asthma/chronic obstructive pulmonary disease. The category “hematological malignancies” includes acuted myeloid leukemia, chronic myeloid leukemia, acute lymphoblastic leukemia, chronic lymphocytic leukemia, non‐Hodgkin lymphoma, Hodgkin lymphoma, multiple myeloma, aplastic anemia, and myelodysplastic syndrome (*n* = 88 total).

BALFs were examined through microscopy (differential cell counts) and standard microbiological methods (staining and culturing for bacteria, including mycobacteria and fungi, as specified by the attending physician). Positive bacterial cultures were measured by counting colony‐forming units per milliliter, followed by identification and susceptibility testing [17]. Incubation duration and conditions were adjusted based on the pathogen type. One microliter of vortexed BAL was spread onto culture plates, including Columbia sheep blood agar and Colistin Nalidixic Acid blood agar for Gram‐positive bacteria, as well as Haemophilus chocolate agar and MacConkey agar for Gram‐negative bacteria. The plates were incubated at 36°C for 2 days [4].

The microbiological diagnostic workup included virus isolation by culture, specific immunofluorescence, and PCR testing for CMV, Epstein–Barr virus, HSV‐1 and HSV‐2, human herpesvirus 6 and 7, and varicella‐zoster virus, as previously reported [18]. Viral PCR testing was performed using validated quantitative laboratory‐developed real‐time PCR assays as reported previously [18]. PCR results were reported as positive when amplification was detected with a cycle threshold (Ct) value < 45. Quantification was based on standard curves ranging from 100 to 1 000 000 copies/mL, generated from serial dilutions of plasmid DNA standards containing the respective viral target genes and included in each analytical run, with viral genome concentrations extrapolated from the corresponding standard curve and reported as copies/mL. Among samples, the median viral load was 4600 copies/mL (IQR, 1000–34 806.5). CMV positivity was defined as the detection of CMV by either virus isolation by culture or CMV PCR. Viral genome concentrations in clinical samples are extrapolated from the corresponding standard curve and reported as copies per milliliter (copies/mL). However, as there is no analysis, stratification, or interpretation based on viral loads in the manuscript, PCR results are consistently used as a binary variable (detected vs. not detected) to define exposure groups and outcomes, without reference to quantitative thresholds, viral load cutoffs, or load–response relationships [18]. Additionally, a respiratory panel multiplex‐NAT (RespiFinder SMART 22 FAST, Pathofinder, Maastricht, The Netherlands) was used to test for viruses and bacteria [19]. This panel detects 18 viruses and 4 bacteria, including Adenovirus; *Bordetella pertussis*; Bocavirus; *Chlamydophila pneumoniae*; Coronaviruses NL63, HKU1, OC43, and 229E; Human Metapneumovirus; Influenza virus A, B, and H1N1; *Legionella pneumophila*; *Mycoplasma pneumoniae*; Parainfluenza viruses 1, 2, 3, and 4; RSV A and B; and Rhinovirus/Enterovirus.

A bacterial infection diagnosis was made based on positive bacteriological findings in the BALF or histologic specimens, or through clinical, laboratory, and radiological evidence strongly indicative of a bacterial infection, according to the discharge summary. The presence of oral flora was identified if at least two of the following bacteria were detected: *Viridans Streptococcus group* (excluding *Streptococcus pneumoniae* and *Enterococcus* spp.), coagulase‐negative *Streptococcus*, *Neisseria* spp., nonhemolytic *Streptococcus* spp., or coryneform bacteria without gram‐negative organisms. A possible bacterial infection was indicated by a positive microbiological diagnostic result. A diagnosis of no bacterial infection was made when another cause for the pulmonary symptoms and/or infiltrates was identified.

CMV infection was defined by the detection of CMV DNA in BALF by PCR. CMV disease was diagnosed using a composite approach requiring all of the following: (i) compatible clinical presentation with respiratory deterioration and/or nonspecific symptoms (e.g., fever, cough, dyspnea), (ii) CT findings suggestive of CMV pneumonitis (e.g., ground‐glass opacities), and (iii) absence of an alternative causative pathogen. We acknowledge that the clinical symptoms are not specific to CMV and were therefore interpreted in combination with radiological and virological findings. Extra‐pulmonary CMV manifestations (e.g., colitis, retinitis, encephalitis) were not systematically assessed due to procedural risks in these critically ill patients. Among the patients classified as CMV disease, all showed elevated liver enzymes suggestive of possible CMV hepatitis, though liver biopsy was not performed.

The attending physicians, in collaboration with infectious disease specialists and respiratory physicians, determined the course of antimicrobial therapy and additional diagnostic or therapeutic measures. The study team did not influence the management of patients.

### 2.4 Primary Endpoint

The primary outcome of the study was the incidence of CMV detected by either detection method in BAL samples from immunocompromised patients and its impact on the clinical course, considering coinfections. Additionally, the study assessed changes in the treatment of CMV‐positive patients following bronchoscopy, including initiation and discontinuation of therapy.

### 2.5 Statistical Analysis

Categorical variables were expressed as frequencies and proportions, while continuous variables were presented as median values with interquartile ranges. Differences among classification groups were assessed using either the Kruskal–Wallis test or Fisher's exact test, depending on the nature of the variables. The association between hospital admission type and CMV infection groups was examined using mixed logistic regression models, while the relationship between the length of hospital stay and CMV infection groups was analyzed using mixed linear regression models. Survival beyond 30 days from BAL was analyzed using the Kaplan–Meier method. To calculate overall 30‐day mortality risk ratios, Cox regressions were employed, incorporating age, sex, type of immunosuppression, infection type, and changes in therapy management. All models included the subject as a random effect to account for cases involving more than one visit. Survival analysis and Cox regressions were conducted for all subjects and stratified by CMV infection. The statistical analysis was performed using the statistical analysis system (SAS Institute Inc., Cary, North Carolina, USA). A significance level of 0.05 was applied to all comparisons.

## 3 Results

### 3.1 Baseline Characteristics

A total of 2666 cases, that represent 2666 visits from 1301 patients, were included in the study. Among them, 235 BALFs from 156 patients had a positive CMV PCR test in BALF, with 98.7% diagnosed with CMV infection and 3 cases (1.3%) classified as CMV disease. These cases were classified as hematological (*n* = 88; 67 patients), solid organ transplantation (*n* = 70; 25 patients), and other causes (*n* = 77; 64 patients, Figure 1). Of the 156 patients, 43 individuals (25.1%), corresponding to 59 visits, had isolated CMV infection, while 114 patients (74.9%), accounting for 176 visits, had CMV infection accompanied by coinfections (fungal *n* = 70, bacterial *n* = 35, or viral *n* = 71). The median age of the 156 patients was 56 years (IQR: 46–65), with 45.5% of the cohort being female (Table 1).

**Figure 1:**
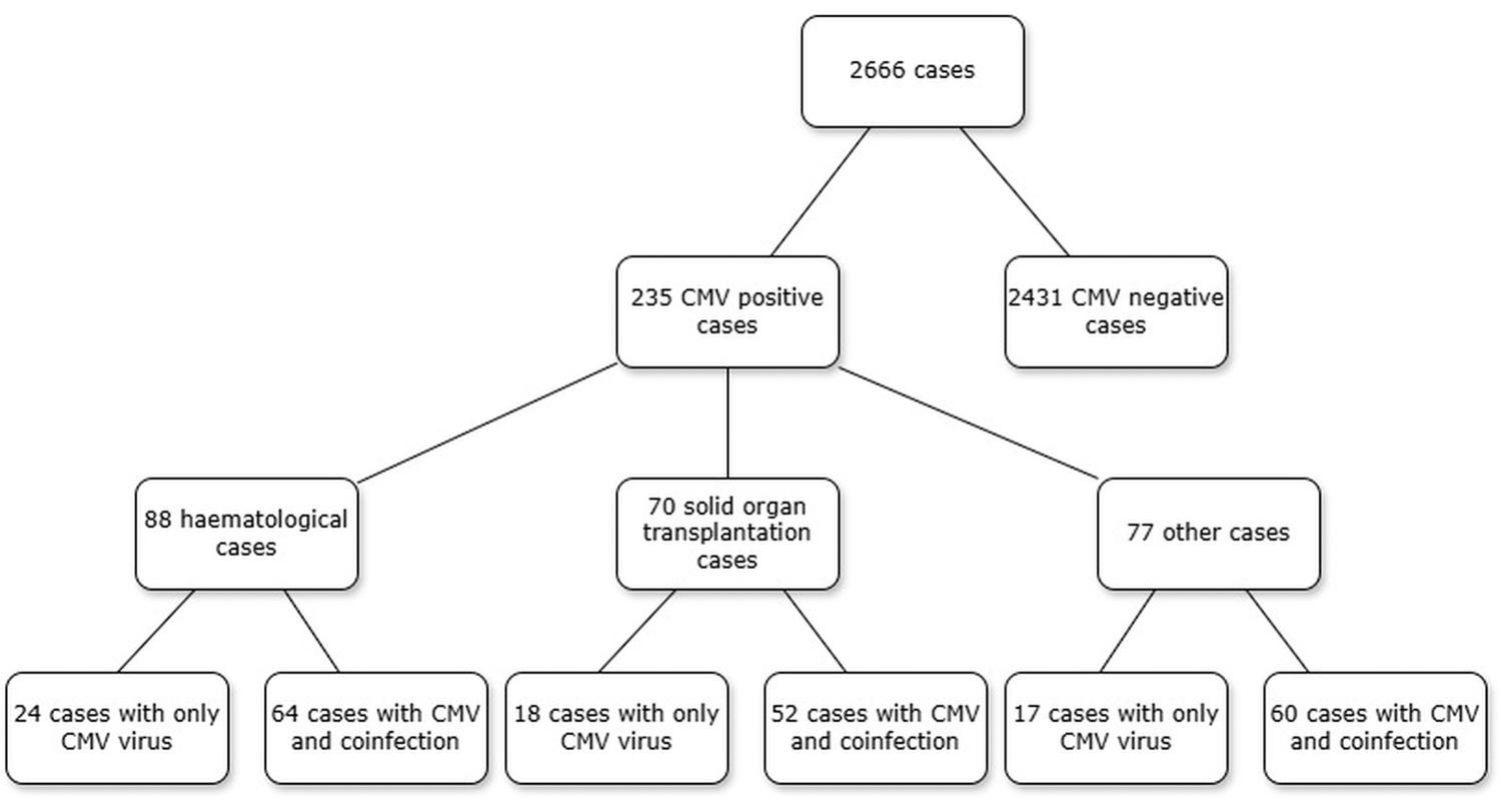
Schematic diagram of 2666 cases, representing 2666 visits from 1301 immunocompromised patients, according to CMV infections.

**Table 1:** Sociodemographic and clinical characteristics of all 1301 immunocompromised patients with and without CMV infection, based on 2666 visits, presented overall and with CMV‐positive cases stratified by immunosuppression group.

Characteristic	CMV‐negative cases (*n *= 2431)	CMV‐positive cases (*n* = 235)	Hematological cases (*n* = 88)	Solid organ transplantation cases (*n* = 70)	Other cases (*n* = 77)	*p* *
Age, years [median (IQR)]	58.0 (46.0–65.0)	56.0 (46.0–65.0)	55.0 (44.3–66.7)	57.0 (46.5–62.0)	57 (46.5–73.0)	0.228
*Sex*						
Female [*n* (%)]	1047 (43.1)	107 (45.5)	29 (33)	35 (50)	43 (55.8)	0.009
Symptoms		201 (85.5)	78 (88.6)	57 (81.4)	66 (85.7)	
Cough [*n* (%)]	1509 (62.1)	139 (59.2)	48 (54.5)	41 (58.6)	50 (64.9)	0.398
Dyspnea [*n* (%)]	751 (30.9)	71 (30.2)	30 (34.1)	17 (24.3)	24 (31.2)	0.403
Sputum [*n* (%)]	709 (29.2)	84 (35.7)	26 (29.5)	24 (34.3)	34 (44.2)	0.143
Fever [*n* (%)]	455 (18.7)	58 (24.7)	22 (25.0)	12 (17.1)	24 (31.2)	0.144
Chest CT scan (n = 180)						
No abnormal radiological finding [*n* (%)]	188 (7.74)	13 (5.5)	5 (6.2)	4 (26.7)	4 (7.0)	0.986
Infiltrate [*n* (%)]	1073 (44.2)	102 (43.4)	61 (75.3)	8 (53.3)	33 (57.9)	< 0.001
Ground‐glass opacity [*n* (%)]	168 (6.9)	19 (8.1)	8 (9.9)	1 (6.7)	10 (17.5)	0.034
Solitary pulmonary nodule [*n* (%)]	162 (6.7)	12 (5.1)	5 (6.2)	2 (13.3)	5 (8.8)	0.584
Interstitial Lung Disease [*n* (%)]	94 (3.9)	6 (2.6)	2 (2.5)	0	4 (7.0)	0.137
Bronchiectasis [*n* (%)]	75 (3.1)	7 (3.0)	3 (3.7)	0	4 (7.0)	0.267
Bronchiolitis [*n* (%)]	53 (2.2)	5 (2.1)	2 (2.5)	0	3 (5.3)	0.264
Pleural effusion [*n* (%)]	48 (2.0)	5 (2.1)	2 (2.5)	2 (13.3)	1 (1.8)	0.801
Atelectasis [*n* (%)]	46 (1.9)	1 (0.4)	1 (1.2)	0	0	0.431
Cavitation [*n* (%)]	28 (1.2)	2 (0.8)	0	1 (6.7)	1 (1.8)	0.548
Lymphadenopathy [*n* (%)]	39 (1.6)	2 (0.8)	1 (1.2)	0	1 (1.8)	0.649
Pleural plaques [*n* (%)]	1 (0.1)	1 (0.4)	0	0	1 (1.8)	0.361
Chest X‐ray (n = 29)						
No abnormal radiological finding [*n* (%)]	48 (2.0)	4 (1.7)	1 (25.0)	3 (33.3)	0	0.118
Infiltrate [*n* (%)]	102 (4.2)	21 (8.9)	3 (75.0)	6 (66.7)	12 (75.0)	0.025
Atelectasis [*n* (%)]	6 (0.3)	2 (0.9)	0	0	2 (12.5)	0.129
Interstitial Lung Diseases [*n* (%)]	7 (0.3)	2 (0.9)	0	0	2 (12.5)	0.361
BAL parameters (n = 132)						
Macrophages % median (IQR)	70.5 (39.0–88.0)	59.0 (25.8–81.3)	66.0 (32.0–84.0)	58.0 (17.0–73.0)	47.8 (26.0–78.8)	0.295
Neutrophils % median (IQR)	11 (3.0–40.0)	24.5 (5.0–61.8)	18.0 (4.0–48.0)	37.0 (6.0–83.0)	27.5 (3.8–60.0)	0.265
Lymphocytes % median (IQR)	5.3 (2.0–13.0)	6.0 (2.0–13.0)	7.0 (3.0–13.5)	4.0 (1.0–10.0)	5.5 (2.0–13.9)	0.209
Bronchoscopy findings						
No abnormal bronchoscopic finding [*n* (%)]	833 (34.3)	71 (30.2)	28 (31.8)	16 (23.5)	27 (36.0)	0.249
Erythema of the mucosa [*n* (%)]	745 (30.7)	65 (27.8)	33 (37.5)	9 (13.2)	23 (30.3)	0.007
Endobronchial secretion [*n* (%)]	491 (20.2)	51 (21.7)	15 (17.0)	16 (23.5)	19 (24.7)	0.425
Pus [*n* (%)]	465 (19.1)	63 (26.8)	16 (18.2)	30 (42.9)	16 (21.3)	0.001
Bloody secretion [*n* (%)]	114 (4.7)	10 (4.3)	9 (10.2)	0	1 (1.3)	0.005
Bronchial or tracheal stenosis [*n* (%)]	47 (1.9)	6 (2.6)	1 (1.1)	4 (5.9)	1 (1.3)	0.137
Endobronchial tumor [*n* (%)]	43 (1.8)	4 (1.7)	0	1 (1.5)	3 (3.9)	0.155
Crusts [*n* (%)]	24 (1.0)	3 (1.3)	1 (1.1)	2 (2.9)	0	0.303
Inflammatory parameters						
Absolute neutrophil counts, g/L median (IQR)	4.8 (2.8–7.4)	4.1 (2.4–6.3)	3.6 (1.8–4.9)	3.4 (2.7–4.9)	6.2 (4.1–10.5)	< 0.001
C‐reactive protein, mg/L median (IQR)	16 (3.3–72.5)	24 (4.3–69.1)	31.0 (8.4–97.6)	4.3 (1.5–32.4)	42.0 (11.4–71.5)	< 0.001
Leukocytes, g/L median (IQR)	7 (4.6–10.2)	6.3 (4.5–8.6)	5.4 (2.8–7.5)	5.9 (4.9–7.1)	7.9 (6.2–11.2)	< 0.001
30‐day mortality, died [*n* (%)]	123 (5.07)	32 (13.62)	17 (19.3)	6 (8.6)	9 (11.7)	0.004
Length of hospital stay, days, median (IQR)	7 (0–23)	7 (0–23)	12.5 (1–36)	0.5 (0.0–8.0)	9 (2–21)	< 0.001
Antimicrobial therapy [*n* (%)]:						
None	909 (37.4)	83 (35.4)	27 (30.7)	34 (48.6)	22 (28.5)	< 0.001
Start	778 (32.0)	87 (37.2)	29 (32.9)	23 (32.9)	35 (45.5)	
Continue	678 (27.9)	60 (25.5)	31 (35.2)	12 (17.1)	17 (22.1)	
Stop	66 (2.7)	5 (2.1)	1 (1.1)	1 (1.3)	3 (3.9)	

* The *χ* ^2^ or Fisher's exact test was used to compare categorical characteristics among immunosuppression groups with CMV infection. The Kruskal–Wallis test was applied to compare continuous characteristics.

### 3.2 Clinical Outcomes

#### 3.2.1 Hospitalization

CMV‐positive cases exhibited a significantly higher probability of requiring hospitalization compared to CMV‐negative cases (OR 1.39, 95% CI: 1.03–1.87, *p* = 0.030). Numerically, the mean duration of hospitalization was higher (22.91 days for CMV‐positive patients versus 19.86 days for CMV‐negative patients, *p* = 0.140, Table 2). In CMV‐positive cases (*n* = 235), hematological patients exhibited a significantly longer mean hospital stay, when compared to the solid organ transplantation and other immunosuppression groups (respectively 25.3 days, 95% CI: 19.9–30.7 days; 9.4 days, 95% CI: 3.7–15.0 days, and 16.5 days, 95% CI: 11.1–21.9 days, *p* = < 0.001; Figure 2). Furthermore, cases with concurrent CMV and bacterial coinfection demonstrated numerically with longer hospitalization duration when compared to those with CMV infection alone or CMV infection with other viral coinfections (25.03 days, 95% CI: 16.5–33.6 days vs. 14.02 days, 95% CI: 7.92–20.1 days vs. 10.20 days, 95% CI: 3.13–17.3 days, respectively, *p* = 0.085) but there was no significant difference in hospitalization duration among these groups (Figure 3).

**Table 2:** CMV infection impact on the length of hospital stay adjusted for immunosuppression group, gender and age (only stationary admissions, *N* = 1694).

Outcome*	Factor	Value	Estimate	StdErr	*p*	Adjusted mean in days	95% CI for mean	*p* †
Length of hospital stay in days	Intercept	—	24.07	2.78	< 0.001			
	CMV infection	Yes	3.05	2.07	0.140	22.91	19.04–26.79	0.140
		No	0			19.86	18.52–21.20	Ref.
	Study group	Hematological	16.25	1.38	< 0.001	32.55	30.06–35.04	< 0.001
		Solid organ Tx	−0.99	1.75	0.570	15.31	12.09–18.52	0.766
		Other	0			16.30	13.75–18.85	Ref.
	Gender	Male	1.04	1.24	0.401	21.91	19.53–29.24	0.401
		Female	0			20.87	18.44–23.29	Ref.
	Age	1 year increase	−0.17	0.04	< 0.001			

*Note:* Estimate = regression coefficient; StdErr = standard error for the estimate; Ref. = reference level for comparison within categorical factors.

* Results of a mixed linear regression model which included the effect of the subject as a random effect.

† *p* value adjusted for multiple comparisons using the Dunnett–Hsu method.

**Figure 2:**
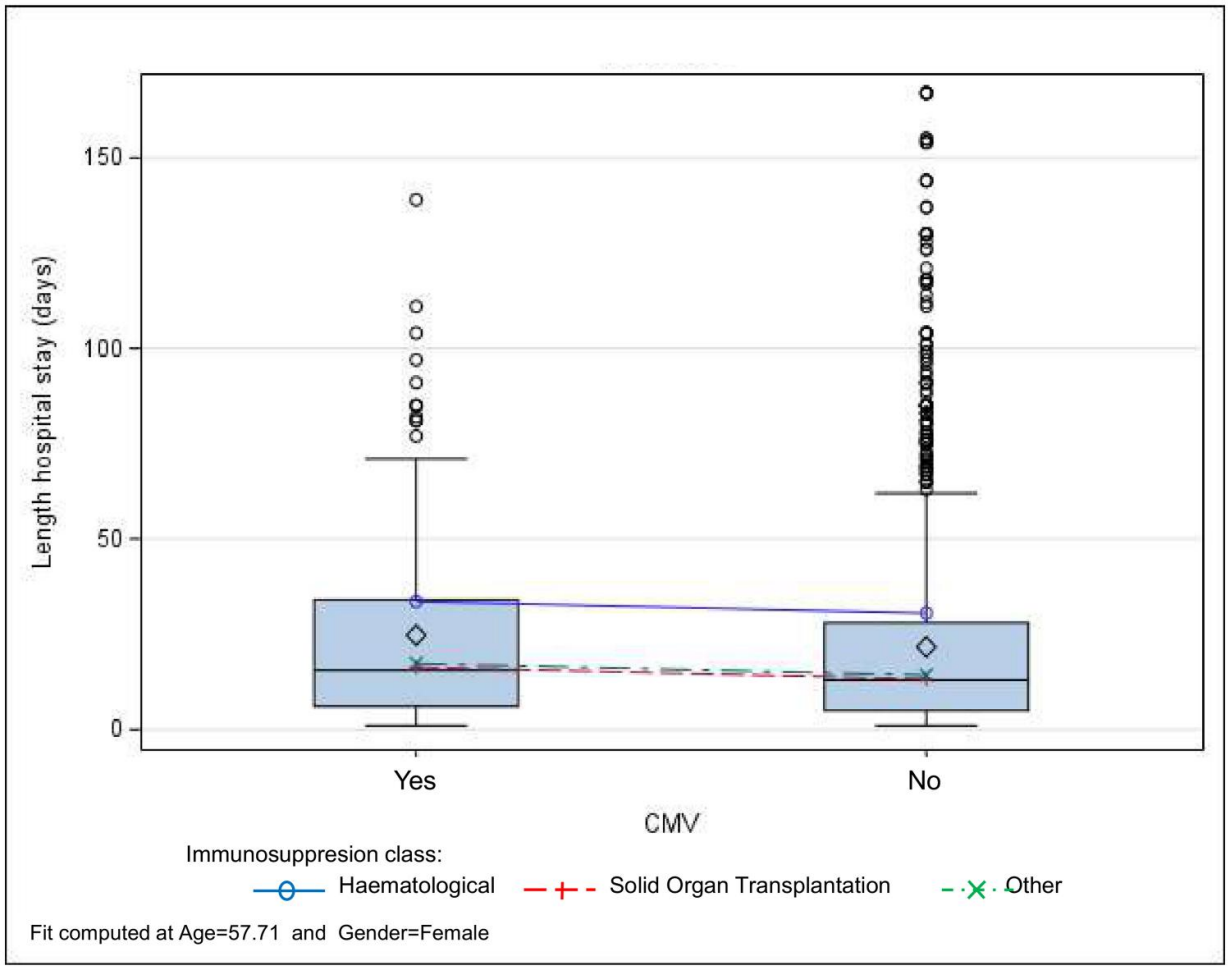
Length of hospital stay among immunosuppressed patients with and without CMV infection, adjusted by immunosuppression group, age, and gender. Box plots show the mean, with whiskers representing the 95% confidence interval. The blue line indicates hematological cases, the red line indicates solid organ transplant cases, and the green line represents other cases.

**Figure 3:**
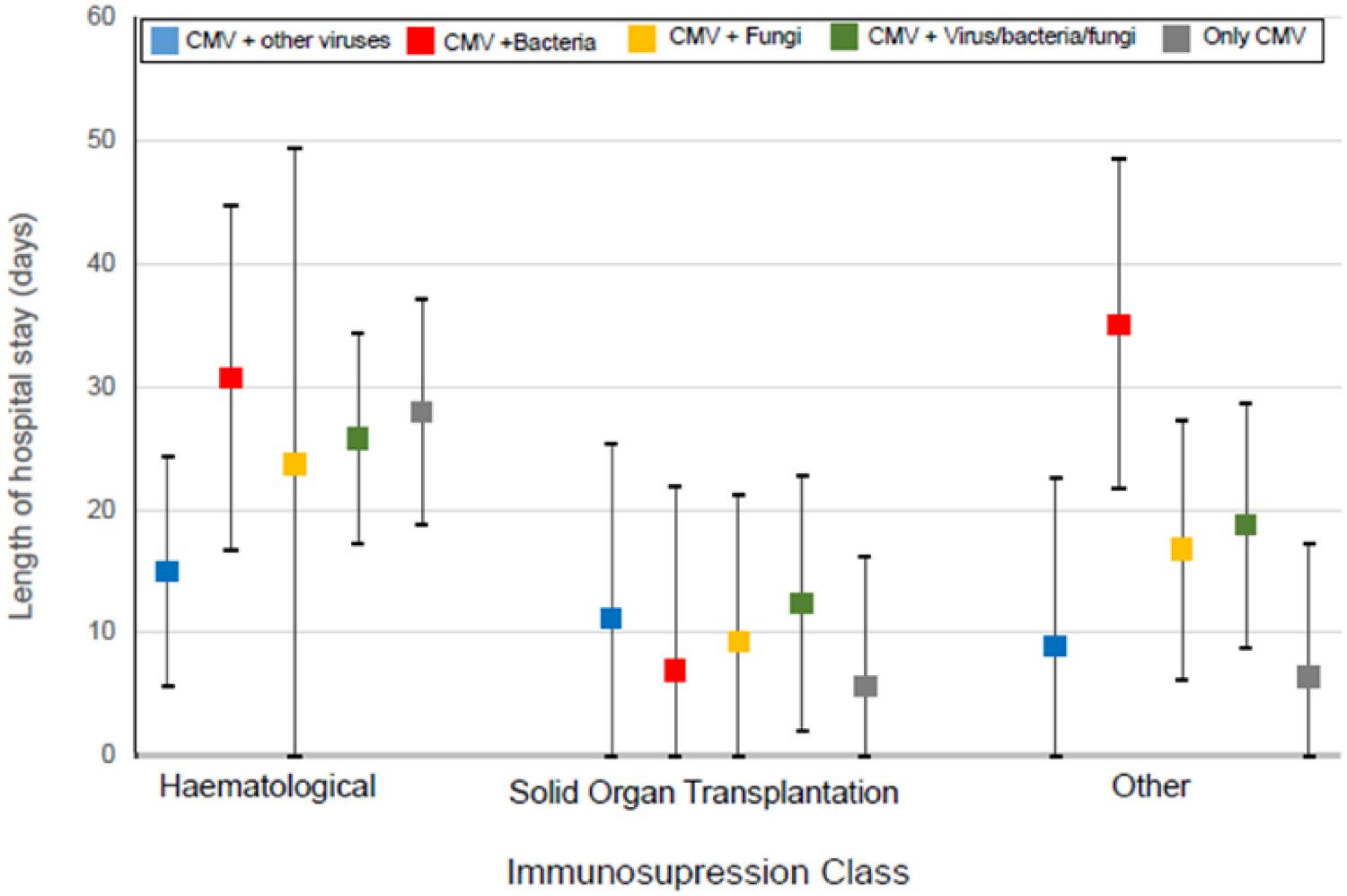
Length of hospital stay for immunosuppressed patients with CMV infection and co‐infection with any other virus, bacteria, or fungi, including *Pneumocystis jirovecii* and *Mycobacteria*, adjusted by immunosuppression group, age, and gender. The square represents the adjusted mean and the whiskers the 95% confidence interval.

#### 3.2.2 30‐Day‐Mortality Rate

The 30‐day mortality was significantly higher in cases with CMV infection compared to those without CMV (13.6% vs. 5.1%, respectively, HR 2.88, 95% CI: 1.88–4.43, *p* < 0.001; Figure 4). 30‐day mortality significantly increased with age (HR 1.02, 95% CI: 1.0–1.03, *p* = 0.002) and numerically with male sex (Table 3). Cases with CMV infection alone tended to be at greater risk of fatal outcomes (*n* = 11, 18.6%) compared to those with bacterial (*n* = 3, 10%) or viral coinfections (*n* = 4, 8.9%, *p* = 0.443).

**Figure 4:**
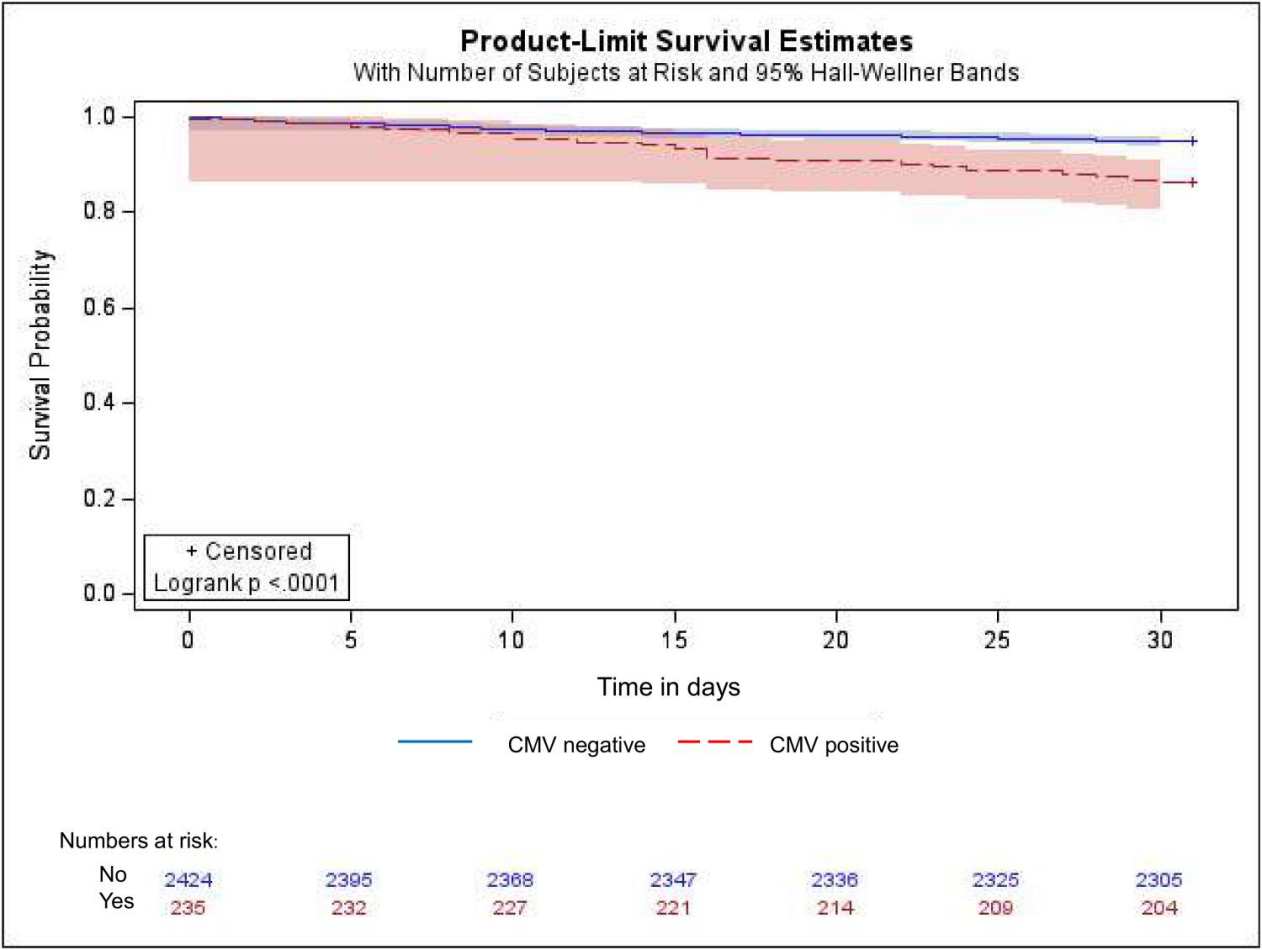
Cumulative probability of 30‐day survival after bronchoscopy in CMV‐positive and CMV‐negative cases.

**Table 3:** Cox regression to determine the 30‐day mortality hazard ratio of CMV positive compared to negative.

Outcome*	Factor	Estimate	StdErr	Hazard ratio (HR)	95% CI for HR	*p* †
30‐day mortality	CMV (Yes vs. No)	1.05	0.22	2.88	1.88–4.42	< 0.001
	Primary composite (Yes vs. No)	0.19	0.17	1.21	0.86–1.69	0.270
	Age (1 yr increase)	0.02	0.01	1.02	1.01–1.03	0.002
	Sex (female vs. male)	−0.21	0.18	0.81	0.57–1.16	0.248

*Note:* Adjusted association by primary composite (change in therapy), age, and gender. Primary composite = included new steroid prescription, steroid augmentation, prescription of other immunosuppressants, immunoglobulins, indication for video‐assisted thoracoscopy surgery and antimicrobial therapy initiation or cessation as therapy management.

* Results of a Cox regression model which included the subject effect as random effect.

† *p* value adjusted for multiple comparisons using the Dunnett–Hsu method.

The type of treatment (therapeutic, prophylactic, and no therapy) was not associated with the 30‐day mortality rate (*p* = 0.142). Among CMV‐positive cases, those who had received prophylactic treatment exhibited a lower mortality rate when compared to those who received CMV treatment (13.9% vs. 42.9%). The mortality rate was similar in cases without treatment and those receiving CMV prophylaxis (12.5% vs. 13.9%).

Among CMV‐positive patients, BAL findings resulted in a change in management in 145 of 235 cases (61.7%). 30‐day mortality did not differ significantly between patients with and without a change in therapy (15.9% vs. 10.0%; *p* = 0.072). Similarly, length of hospital stay was comparable between groups (mean 17.45 ± 22.53 days vs. 16.57 ± 22.56 days; *p* = 0.075).

#### 3.2.3 Symptoms and Bronchoscopic Findings

In 85.5% of cases, patients exhibited symptoms, with a higher prevalence in patients with CMV and coinfections, when compared to those with a CMV monoinfection (93.9% vs. 76.3%; *p* = 0.012). Cough was frequently observed in CMV‐infected patients (59.2%), particularly in those with coinfections (bacterial, fungal, or other viral) compared to isolated CMV infection (77.3% vs. 32.2%, respectively, *p* < 0.001). Similarly, expectoration was more common in CMV‐infected patients with coinfections compared to CMV monoinfection (13.6% vs. 45.5%; *p* = 0.001). Putrid secretions were identified during bronchoscopy in 39.4% of cases with CMV and coinfections, when compared to 13.6% cases with CMV monoinfection (*p* = 0.026). Additionally, in 30% of all CMV‐positive cases, bronchoscopy revealed no abnormal bronchoscopic finding, with no significant difference between cases with CMV monoinfection and cases with CMV and coinfections (44.1% vs. 25.8%; *p* = 0.080).

#### 3.2.4 BALF and Inflammatory Parameters

A difference in BALF cytology was observed between cases with CMV monoinfection and those with CMV and coinfections. In cases with CMV monoinfection, a higher percentage of macrophages was observed in all three immunocompromised groups, compared to cases with CMV with coinfections (75.5%, IQR: 46–84 vs. 37.5%, IQR: 11.5–67; *p* = 0.008). In contrast, a higher percentage of neutrophils was observed in BALF of cases with CMV and coinfections, when compared to cases with CMV monoinfection (49.5%, IQR: 15.5–82.5; vs. 7.5%, IQR 2–36 *p* = 0.002).

Among the serum parameters, leukocytes and absolute neutrophil counts did not show a statistically significant difference between CMV monoinfection and CMV coinfection. On the other hand, CRP levels were significantly higher in cases with CMV and coinfections, when compared to those with CMV monoinfection (35.1 mg/L, IQR: 10.4–91.9 vs. 8.5 mg/L, IQR: 3.4–32; *p* = 0.0001).

#### 3.2.5 CT Findings

From all the CMV‐positive cases, CT imaging was performed in 76.6% (*n* = 180) of cases. Among these, 5.5% exhibited no abnormal radiological findings on CT, and there was no significant difference in the rate of no abnormal radiological findings in CT among CMV patients, without and with concomitant infection (8.47% vs. 3%, respectively, *p* = 0.169). The most frequently observed pathological finding was pulmonary infiltrates, detected in 43.4% of CMV‐positive cases. This finding was present in both patients with CMV monoinfection and those with coinfections, with no statistically significant difference between the groups (37.3% vs. 48.5%; *p* = 0.246). On the other hand, there was statistically significant difference among the three immunocompromised subgroups, regarding the prevalence of infiltrates (25.9% vs. 3.4% vs. 14%; in the hematological, solid organ transplant, and other groups, respectively, *p* < 0.001).

#### 3.2.6 Incidence of Concomitant Bacterial, Fungal, and Viral Infections

In 176 CMV‐positive cases (74.9%), a concurrent bacterial, fungal, or viral coinfection was observed. Approximately one‐third of CMV‐positive cases (34.5%, *n* = 81), while only 2,8% (*n* = 69) of CMV negative cases demonstrated a viral coinfection (*p* < 0.001).

Among the total 2666 cases analyzed, the most frequently detected viral pathogens were Rhinovirus/Enterovirus (12.4%) and HSV type 1/2 (4.5%), with a higher prevalence in CMV‐positive cases. Specifically, Rhinovirus/Enterovirus coinfection was observed in 20% of CMV‐positive cases compared to 11% in CMV‐negative cases (*p* = 0.012), while HSV was observed in 7.7% of CMV‐positive cases compared to 4.2% of CMV‐negative cases (*p* = 0.022). Furthermore, concomitant viral and bacterial coinfections were significantly more frequent in CMV‐positive than in CMV‐negative cases (35.7% vs. 8.1%; *p* < 0.001).

Bacterial coinfections were detected in 33% (*n* = 78) of CMV‐positive cases, whereas only 24% of CMV‐negative cases (*p* = 0.006). Among the total 2666 cases, the most frequently observed bacterial infections were caused by *Pseudomonas aeruginosa* (4.8%). Among CMV‐positive cases with bacterial coinfection (*n* = 30), 20% were coinfected with *P. aeruginosa*.

Fungal coinfections were observed in 35.9% (*n* = 84) of CMV‐positive cases, and in only 20% (*n* = 486) of CMV‐negative cases (*p* < 0.001). The most prevalent fungal infection was caused by *Candida* species (15%), particularly *Candida albicans* (10%), in both CMV‐positive and CMV‐negative cases. However, a higher prevalence of *Candida* infection was observed in CMV‐positive cases compared to CMV‐negative cases (26% vs. 14%; *p* < 0.001). Additionally, *Pneumocystis jirovecii* coinfection was observed in 2.1% of all 2666 cases. Among CMV‐positive cases with fungal coinfection, 17% were coinfected with *P. jirovecii*.

## 4 Discussion

This study represents the most extensive prospective analysis of immunocompromised patients undergoing BAL, integrating a thorough diagnostic assessment with a longitudinal follow‐up over 7 years. It includes all major categories of immunocompromised individuals and examines CMV incidence in this population while considering the role of coinfections and clinical outcomes.

Among the 2666 immunocompromised cases, 235 cases from 156 patients tested positive for CMV in BALF, with 98.7% classified as CMV infection and 1.3% as CMV disease. Previous studies on smaller cohorts of immunocompromised patients have shown CMV detection in BALF to be a frequent finding, with reported incidence rates ranging between 6% and 44% [20, 21, 22, 23, 24, 25]. These variations may be attributed to differences in patient subgroups, BALF sampling timing in relation to transplantation or immunosuppressive therapy, and the presence or absence of clinical or radiological abnormalities. In our study, all patients suspected of LRTIs underwent diagnostic bronchoscopy, emphasizing the critical role of fiberoptic bronchoscopy in this setting. Up to date, no other study has comprehensively analyzed CMV‐BALF findings in immunocompromised patients with suspected LRTI.

CMV‐positive cases had a significantly higher probability of hospitalization compared to CMV‐negative cases (OR 1.39, 95% CI: 1.03–1.87, *p* = 0.030). CMV infection was associated with a numerically longer duration of hospitalization, although this association did not reach statistical significance (22.91 vs. 19.86 days, *p* = 0.1402), suggesting that other clinical factors may contribute to hospitalization duration. Among different patient groups, hematologic patients experienced the longest hospital stays compared to solid organ transplant recipients and other immunocompromised groups (25.3 days, 95% CI: 19.9–30.7 days, vs. 9.4 days, 95% CI: 3.7–15.0 days, vs. 16.5 days, 95% CI: 11.1–21.9 days, respectively, *p* = < 0.001). No other study has extensively studied the duration and probability of hospitalization among different immunocompromised groups with CMV. These findings suggest that the underlying cause of immunosuppression plays a crucial role in clinical outcomes [26].

In this study, CMV infection was associated with a significantly higher 30‐day mortality compared to CMV‐negative patients (*p* < 0.001). This finding align with a previous study of 114 HIV‐positive patients, where CMV detection in BAL was linked to increased mortality at 3 and 6 months [27]. Additionally, another study reported that immunocompromised patients with LRTI due to CMV exhibit higher mortality rates, emphasizing the potential utility of BAL as a less invasive diagnostic method for early CMV detection [28]. In a previous study, CMV infection has been associated with an increased risk of bronchiolitis obliterans and mortality in transplant recipients [29]. These findings highlight the significant impact of CMV infection on patient outcomes and reinforce the importance of early detection and management.

CMV‐positive patients who initiated therapeutic treatment after bronchoscopy without prior prophylaxis exhibited a higher mortality risk. Although this finding should be interpreted with caution, it likely reflects underlying differences in baseline clinical status, as patients receiving prophylaxis were generally managed according to routine post‐transplant control protocols and were therefore more stable, whereas those started on therapeutic treatment were, by definition, already symptomatic and more severely ill, prompting bronchoscopy and initiation of therapy. Several retrospective studies have shown that late‐onset CMV disease is common in lung transplant recipients, even after receiving a CMV prophylaxis for 3–6 months [30, 31]. This “late‐onset CMV disease,” typically defined as infection occurring after the prophylactic period ends, has been associated with a higher risk of graft failure following kidney transplantation and increased mortality in liver transplant recipients [32, 33]. The absence of prophylaxis may be linked to worse clinical outcomes compared to those who received prophylactic care, particularly in the initial months following immunosuppression and late‐onset CMV infection remains a critical concern [34, 35, 36]. While prevention strategies are biologically plausible and clinically appealing, randomized controlled trials are ultimately required to determine the true efficacy of CMV prophylaxis and clarify its impact on outcomes in immunocompromised populations.

Our study revealed a high prevalence of coinfections in CMV‐positive patients, with 74.9% exhibiting concurrent bacterial, fungal, or viral infections. The incidence of CMV‐viral, CMV‐bacterial, and CMV‐fungal coinfections was approximately 30% each, with the most frequently detected viral pathogens being Rhinovirus/Enterovirus (12.4%) and HSV type 1/2 (4.5%). These findings align with a previous prospective study, which demonstrated that immunocompromised patients are at an increased risk for LRTI, with rhinovirus/enterovirus being the most frequently detected virus [4].

The elevated rate of concomitant infections in CMV‐positive patients reflects the underlying immunocompromised state, with CMV reactivation serving as both a marker of immune vulnerability and a contributor to secondary infections through its immunosuppressive effects. CMV exerts its immunosuppressive effects through multiple mechanisms, including immune evasion, impairment of natural killer (NK) cell function, T‐cell dysfunction, cytokine modulation, and hematopoietic dysfunction. By downregulating major histocompatibility complex class I and II molecules, CMV reduces antigen presentation, allowing the virus to evade T‐cell recognition [6, 37]. At the same time, it disrupts NK cell function, weakening innate immunity. Chronic CMV infection leads to sustained immune activation and T‐cell memory inflation, resulting in an overrepresentation of CMV‐specific T cells at the expense of responses to other infections [38]. CMV also alters cytokine production, suppressing inflammatory responses needed for viral clearance and producing virus‐encoded IL‐10 [39] and TGF‐β [40], which inhibit immune activation. In addition, it affects bone marrow function, causing lymphopenia and impaired immune reconstitution, further increasing susceptibility to opportunistic infections [38]. These immunosuppressive effects may explain why CMV‐positive patients in our study experienced a higher incidence of bacterial, fungal, and viral coinfections. Clinically, this can lead to worse outcomes, prolonged hospitalization, and increased mortality due to secondary infections and immune dysregulation.

There are few limitations to consider, including the fact that all the patients underwent a bronchoscopy with BAL, but without transbronchial biopsy. While BAL is a widely used and less invasive diagnostic tool, a transbronchial biopsy could have provided histopathological confirmation, helping to differentiate between CMV infection and CMV disease more precisely. However, given the fragile nature of this patient population, performing a transbronchial biopsy carries a higher risk of complications, which may limit its feasibility in routine clinical practice. Additionally, this study does not follow a case‐control design, meaning the observed associations may be influenced by unmeasured confounding. Variations in patient characteristics—such as the indication for transplantation, degree of immunosuppression, or presence of comorbidities—could independently affect both CMV incidence and clinical outcomes. However, the potential impact of these confounders is likely minimized by the relative homogeneity of the patient cohorts with respect to baseline characteristics and treatment protocols, which reduces the risk of systematic bias and strengthens the overall validity of the findings. Another limitation is that this study was conducted before the SARS‐CoV‐2 pandemic, and the data reflect pre‐pandemic prevalence rates of CMV infection and its coinfections.

Another limitation of our study is the lack of systematic evaluation for extra‐pulmonary CMV manifestations, including CMV colitis/gastritis, retinitis, hepatitis, and encephalitis. Some patients presented with diarrhea, but definitive diagnosis of CMV colitis required endoscopy with biopsy, which was not feasible due to procedural risks in these critically ill patients. Similarly, ophthalmologic examinations, including fundoscopy for CMV retinitis, cerebrospinal fluid analysis for encephalitis, and liver biopsies for suspected CMV hepatitis were not performed, given the patients' severe condition, coagulopathy risks, and need for intensive organ support.

Despite these few limitations, our study has several strengths, as it represents the largest prospective analysis of immunocompromised patients undergoing BAL, providing comprehensive insights into the incidence and clinical impact of CMV in this high‐risk population.

## 5 Conclusion

These findings highlight the impact of CMV infection on hospitalization rates and mortality, emphasizing the need for preventive strategies such as prophylactic treatment. Coinfections, particularly bacterial and fungal, play a critical role in clinical outcomes. Immunocompromised patients are at higher risk for CMV infection, and its presence does not preclude simultaneous coinfection with other respiratory viruses, bacteria, or fungi. Furthermore, BAL plays a crucial role in guiding clinical management, leading to changes in most cases, which can improve survival in immunosuppressed patients. These findings emphasize the need for thorough diagnostic and treatment strategies to effectively manage CMV and associated infections.

## Author Contributions

Daiana Stolz, Eleni Papakonstantinou, and Theodora Zouvani conceptualized and designed the study. Daiana Stolz and Theodora Zouvani were responsible for the recruitment of participants. Daiana Stolz, Eleni Papakonstantinou, Meropi Karakioulaki, Theodora Zouvani, Kathleen Jahn, and Karoline Leuzinger collected the data and interpreted the results. Leticia Grize and Daiana Stolz did the statistical analysis. Daiana Stolz, Theodora Zouvani, Meropi Karakioulaki, and Leticia Grize wrote the manuscript. All authors contributed to the manuscript review and approved the submitted draft. All authors had full access to all the data in the study and accept responsibility for the decision to submit for publication. Daiana Stolz and Leticia Grize have accessed and verified all the data in the study. All authors are accountable for all aspects of this study and ensure that questions related to the accuracy or integrity of the data are appropriately investigated and resolved.

## Funding

The authors have nothing to report.

## Ethics Statement

The study was approved by the Institutional Review Board of the University Hospital Basel, Switzerland (EKBB05/06).

## Consent

Informed consent was obtained from all participants.

## Conflicts of Interest

The authors declare no conflicts of interest.

## Supporting information


Supporting File 1

## Data Availability

The data that support the findings of this study are available from the corresponding author upon request.
