# Supplementary material for: Cytomegalovirus in Bronchoalveolar Lavage Fluid: A Predictor of Outcome in Immunocompromised Patients
Source: J Med Virol. 2026 Sep 28;98(10):e71163. doi: 10.1002/jmv.71163 (PMC13620442; doi:10.1002/jmv.71163)
Supplement: Supplementary file 1 [file JMV-98-e71163-s001.pdf]

CMV Infections and co-infections	Antibacterial Therapy	Antiviral Therapy	Antifungal Therapy	All pathogens (all combinations)	Nr of Cases	% out of 140 cases with a change in therapy management
Only CMV	None	therapy	therapy	No pathoges, only CMV	1	0.7143
	None	Start	None	No pathoges, only CMV	7	5.0000
	Start	None	None	No pathoges, only CMV	6	4.2857
	Start	Start	None	No pathoges, only CMV	1	0.7143
	Start	Continue	None	No pathoges, only CMV	1	0.7143
	Start	Continue	Continue	No pathoges, only CMV	3	2.1429
	Continue	None	None	No pathoges, only CMV	2	1.4286
	Continue	Start	None	No pathoges, only CMV	1	0.7143
	Continue	Start	Continue	No pathoges, only CMV	2	1.4286
	Stop	Start	Continue	No pathoges, only CMV	1	0.7143
CMV + other virus	None	Start	None	No pathoges, only CMV	2	1.4286
				HSV1	1	0.7143
				Rhino-Enterovirus	2	1.4286
	Start	None	None	Coronavirus	1	0.7143
				RSV A	1	0.7143
				Rhino-Enterovirus	4	2.8571
				Rhino-Enterovirus & HSV-1	1	0.7143
				humanes Metapneumovirus	1	0.7143
	Start	None	Continue	HSV-1	1	0.7143
	Start	Start	None	HSV1	1	0.7143
	Start	Continue	None	EBV	1	0.7143
	Start	Continue	Continue	Rhino-Enterovirus	1	0.7143
	Continue	Start	Continue	HHV6 & EBV	1	0.7143
	Continue	Continue	None	Adenovirus & Parainfluenza4	1	0.7143
	Stop	None	None	Coronavirus & Rhino-Enterovirus	1	0.7143
				Rhino-Enterovirus	1	0.7143
CMV + bacteria	Start	None	None	Capnocytophaga sputigena	1	0.7143
				E.coli	1	0.7143
				Haemophilus influenzae	2	1.4286
				Koagulase-negative Staphylokokken	1	0.7143
				Moraxhella catarrhalis	1	0.7143
				Mycobacterium tuberculosis	1	0.7143
				Pneumocystis jiovecii	4	2.8571
				Pseudomonas aeruginosa	5	3.5714
				Staphylococcus aureus & Stenotrophomonas maltophilia	1	0.7143
	Start	None	Continue	Acinetobacter baumanii	1	0.7143
	Start	Continue	Continue	Enterokokken	1	0.7143
				Stenotrophomonas maltophilia	1	0.7143
				Streptococcus pneumoniae	1	0.7143
	Continue	Start	Continue	Enterokokken	1	0.7143
CMV + fungi	None	None	Start	Aspergillus	1	0.7143
				Candida albicans	1	0.7143
				Candida albicans & Aspergillus & Aspergillus fumigatus	3	2.1429
	None	Start	None	Candida albicans	1	0.7143
				Candida albicans	3	2.1429
				Candida albicans & Candida nonalbicans	1	0.7143
	Start	Continue	Continue	()	1	0.7143
	Continue	None	None	Candida dubliensis	2	1.4286
				Candida nonalbicans & Aspergillus & Aspergillus fumigatus	1	0.7143
				Penicillium species	1	0.7143
	Continue	None	Start	Aspergillus & Aspergillus fumigatus	1	0.7143
	Continue	None	Continue	Candida albicans & Candida glabrata & Aspergillus	1	0.7143
	Stop	Continue	None	Aspergillus	1	0.7143
CMV + other virus + bacteria + fungi	None	None	Start	H1N1	1	0.7143
				HSV2 & Aspergillus	1	0.7143
				Parainfluenza1 & Aspergillus	1	0.7143
	None	Start	None	Enterobacteriaceae & humanes Metapneumovirus & Candida nonalbicans	1	0.7143
				HSV1 & Candida albicans	1	0.7143
				Influenza A & Candida albicans	1	0.7143

				Rhino-Enterovirus & HSV1 & Candida albicans	1	0.7143
				Staphylococcus aureus & HSV 2	1	0.7143
	None	Start	Continue	HSV1 & Candida albicans & Candida nonalbicans & Aspergillus & Aspergillus fumigatus	1	0.7143
	Start	None	None	Corynebacterium pseudodiphte & Influenza B	1	0.7143
				Corynebacterium pseudodiphte & Rhino-Enterovirus & Candida albicans	1	0.7143
				HSV1 & Candida albicans	1	0.7143
				Haemophilus influenzae & Parainfluenza 3 & HSV1	1	0.7143
				Haemophilus influenzae & Rhino-Enterovirus	1	0.7143
				Haemophilus influenzae & Rhino-Enterovirus & Candida albicans & Aspergillus & Aspergillus fumigatus	1	0.7143
				Influenza A & Candida albicans	1	0.7143
				Moraxhella catarrhalis & Enterobacteriaceae & Rhino-Enterovirus	1	0.7143
				Pneumocystis jiovecii & Candida albicans	4	2.8571
				Pneumocystis jiovecii & Candida nonalbicans	1	0.7143
				Pneumocystisnjiovecii & RSV A	1	0.7143
				Pneumocystis jiovecii & Rhino-Enterovirus	2	1.4286
				Pseudomonas aeruginosa & Candida albicans	1	0.7143
				Pseudomonas aeruginosa & Klebsiella pneumoniae & H1N1	1	0.7143
				Pseudomonas aeruginosa & Pneumocystis jiovecii & Candida albicans & Aspergillus	1	0.7143
				Pseudomonas aeruginosa & Rhino-Enterovirus	1	0.7143
				Staphylococcus aureus & Pseudomonas aeruginosa & Aspergillus	1	0.7143
				Staphylococcus aureus & Pseudomonas aeruginosa & Candida albicans	1	0.7143
				Streptococcus pneumoniae & Candida nonalbicans	1	0.7143
				E.coli & Candida albicans	1	0.7143
				Enterokokken & Rhino Enterovirus & Aspergillus & Aspergillus fumigatus & Aspergillus nidulans	1	0.7143
	Start	None	Continue	atypische Mycobacterien & Aspergillus	1	0.7143
	Start	Start	None	E.coli & Coronavirus & Rhino Enterovirus	1	0.7143
				Enterobacteriaceae & Serratia marcescens & HSV 1 & Candida albicans & Candida krusei	1	0.7143
				Staphylococcus aureus & Rhino Enterovirus & HSV1	1	0.7143
	Start	Continue	None	Pneumocystis jiovecii & Candida albicans	1	0.7143
				Pneumocystis jiovecii & Candida albicans & Aspergillus & Aspergillus niger	1	0.7143
				Pseudomonas aeruginosa & Rhino Enterovirus	1	0.7143
				Pseudomonas aeruginosa & Rhino Enterovirus & Bocavirus	1	0.7143
	Start	Continue	Continue	Haemophilus influenzae & Rhino Enterovirus	1	0.7143
				Pseudomonas aeruginosa & Rhino Enterovirus	1	0.7143
				Pseudomonas aeruginosa & Rhino Enterovirus & Aspergillus	1	0.7143
				Pseudomonas aeruginosa & Serratia marcescens & Candida krusei	1	0.7143
	Continue	None	None	Coronavirus & Coronavirus OC43	1	0.7143
				Koagulase-negative Staphylokokken & Candida albicans & Candida dubliensis	1	0.7143
	Continue	None	Start	Enterokokken & Lactobacillus & HSV 1	1	0.7143
	Continue	Start	None	Serratia marcescens & Citrobacter freudii & HSV 1	1	0.7143
	Continue	Start	Start	Parainfluenza3 & Aspergillus & Aspergillus fumigatus	1	0.7143
	Continue	Continue	None	RSV B & Candida albicans & Aspergillus & Aspergillus fumigatus	1	0.7143
				Rhino-Enterovirus	1	0.7143
	Stop	None	None	Rhino Enterovirus & Penicillium species	1	0.7143
